# Imaging-based assessment of fracture stability does not reliably predict outcomes in patients with two-part proximal humeral fractures and may lead to unnecessary surgeries

**DOI:** 10.1530/EOR-2026-0043

**Published:** 2026-05-01

**Authors:** Stig Brorson

**Affiliations:** ^1^ Centre for Evidence-Based Orthopaedics, Department of Orthopaedic Surgery, Zealand University Hospital, Køge, Denmark; ^2^ Department of Clinical Medicine, University of Copenhagen, Copenhagen, Denmark

**Keywords:** shoulder fractures, proximal humeral fractures, fracture stability, non-surgical treatment, evidence-based orthopedics

## Abstract

- Two-part surgical neck fractures are the most common displaced proximal humeral fractures in the elderly. Most fractures can be categorized into varus-impacted or medially translated fracture patterns.
- The natural healing process often involves secondary displacement and partial resorption of the humeral head, but these changes are poorly correlated with shoulder function and patient satisfaction.
- Randomized trials have been unable to identify any benefits from surgery but report a high proportion of implant-related complications.
- In large prospective cohort studies, patients aged 60 or older with two-part surgical neck fractures treated non-operatively report shoulder function and quality of life close to the background population six months post-injury.
- Evidence-based and eminence-based approaches to interventions for osteoporotic proximal humeral fractures appear to collide.

## The epidemiology of two-part surgical neck fractures

**Figure 1:**
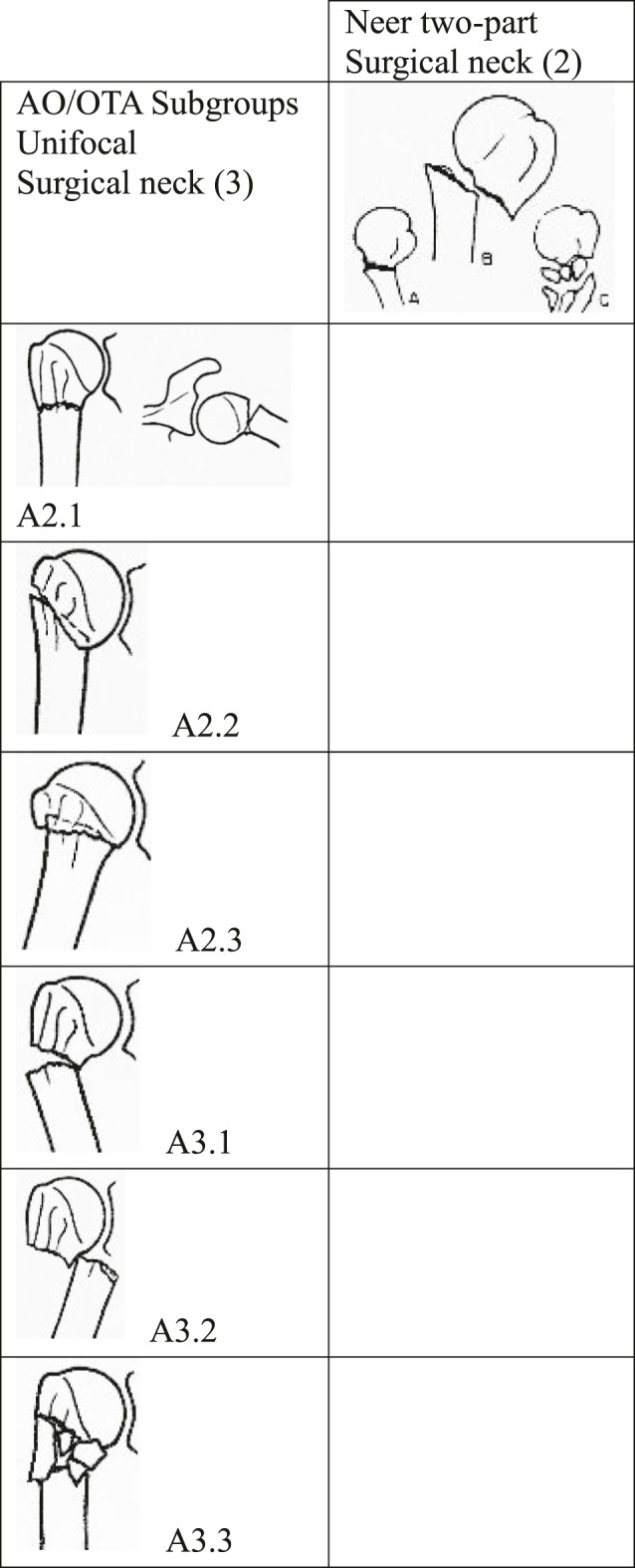
Two-part surgical neck fractures, as outlined by the Neer and AO classifications, highlight the challenges associated with translation. The AO classification is more satisfactory from a pathoanatomical perspective, but lacks a definition of displacement. The Neer classification clearly defines displacement but does not capture varus and valgus displacements in the classification categories.

Proximal humeral fractures are the third most common non-vertebral osteoporosis-related fractures, surpassed only by proximal femur and distal radius fractures. The prevalence among females aged 60 or older has been reported to be as high as 500/100,000/year (1). Large epidemiological studies classifying proximal humeral fractures are scarce. Surgical neck fractures are most commonly classified using the Neer classification (2) or the AO classification (3) (Fig. 1). Unfortunately, the categories cannot be readily translated between the two systems (4). While the Neer classification clearly defines displacement as at least 1 cm or 45 degrees of angulation between the head and shaft, it does not differentiate between varus and valgus displacements. On the other hand, the AO classification distinguishes between varus and valgus displacements, but it lacks a definition of displacement. The relevant categories in both systems are shown in Fig. 1. In his 1970 paper, Neer proposed a distinction among two-part surgical neck fractures with angulation, separation, or comminution (2). These subcategories, however, have not been adopted for clinical or scientific use.

In the 2018 revision of the AO classification (5), displacement was included as a ‘universal identifier’ to incorporate the Neer classification into fracture descriptions. However, without a clear definition of displacement, this change holds limited value.

A prospective cohort study of 1,027 consecutive proximal humeral fractures reported that 28% of all fractures were Neer two-part surgical neck fractures (6). Six subgroups within the AO classification contain bifocal fracture patterns involving the surgical neck: 27% of all proximal humeral fractures were classified as A2 subgroups (A2.1 (9%), A2.2 (13%), and A2.3 (5%)) and 20% were classified as A3 subgroups (A3.1 (3%), A3.2 (13%), and A3.3 (4%)). An ongoing prospective cohort study (7) classified a consecutive series of 850 patients aged 60 or older with proximal humeral fractures. They found 371 (44%) Neer two-part fractures. According to the AO classification, 337 (40%) were AO A2 fractures and 105 (12%) were AO A3 fractures (unpublished interim data). Population characteristics and classification practices may explain differences in the distribution of categories. In the literature, the prevalence of displaced fractures, as defined by Neer, has been reported to range from 15 to 64% across populations (8). However, Neer two-part surgical neck fractures remain the most common pattern of displaced fractures.

Classification data should be interpreted with caution, as substantial inter- and intraobserver variation is a well-known drawback of both classification systems (9). There is no gold standard for classifying these fractures, and the addition of advanced imaging modalities has not improved observer agreement. In the two epidemiological studies above, one senior surgeon performed all classifications.

## Patterns of displacement in two-part surgical neck fractures

**Figure 2:**
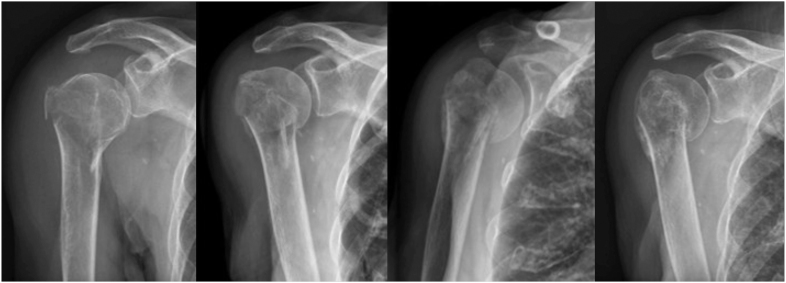
Example of the natural course of a surgical neck fracture. Initially minimally displaced, the fracture collapsed into varus during healing. Anterior–posterior views at admission, 2 weeks, 6 weeks, and 6 months.

The most common patterns of displacement are varus impaction and medial translation. The pattern of displacement depends on the deforming muscular forces acting on the fragments and on bone quality. The pull from the pectoralis major tends to translate the shaft medially and anteriorly. The deltoid pulls the shaft superiorly, while the supraspinatus, attached to the greater tuberosity, rotates the humeral head, thereby diminishing the acromiohumeral distance. Impaction and varus collapse of the humeral head commonly occur in osteopenic bone. Varus impaction can present as initial or secondary displacement (Fig. 2).

**Figure 3:**
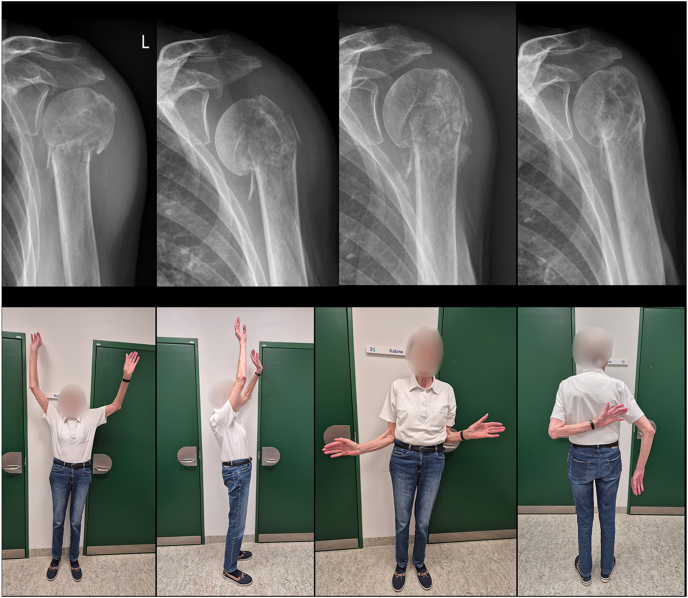
An 82-year-old female with a minimally displaced surgical neck fracture. Varus collapse of the humeral head occurred within the first two weeks post-injury. Partial humeral head resorption was observed during healing. The patient achieved pain-free shoulder function. Anterior–posterior radiographs were taken at admission, 2 weeks, 6 weeks, and 6 months. Photographs were taken after 6 months.

The impaction of the humeral shaft into the humeral head may concern the surgeon, as we usually regard secondary displacement as a sign of instability. However, some degree of collapse or resorption of the humeral head can be expected. Solid healing is usually the result, even if marked malunion develops (Fig. 3). This can be considered the natural healing of the fracture in osteopenic bone. While follow-up to monitor progress in rehabilitation and pain reduction is indicated, surgery based solely on radiographic findings may result in overtreatment and complications if locking plates are used to counter the expected varus collapse or translation of the humeral head.

**Figure 4:**
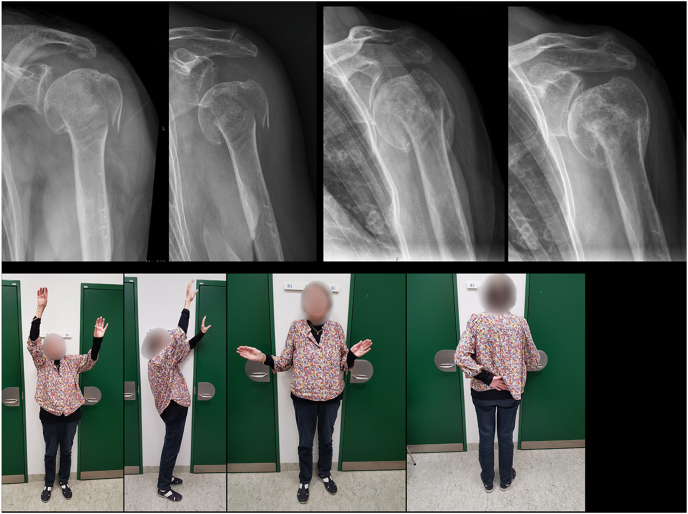
A 90-year-old female with a centrally impacted surgical neck fracture. During healing, the humeral shaft became further impacted. Although the fracture healed with shortening, the patient achieved pain-free shoulder function. The humeral head became better centered in the glenoid during rehabilitation and as healing progressed. External rotation remained restricted. Early signs of glenohumeral osteoarthritis were present. Anterior–posterior radiographs were taken at admission, 2 weeks, 6 weeks, and at 6 months. Photographs were taken at 6 months.

In some cases, the osteopenic humeral head offers limited resistance from the cancellous bone and is compromised by the powerful deltoid muscle, leading to central shaft impaction (Fig. 4).

**Figure 5:**
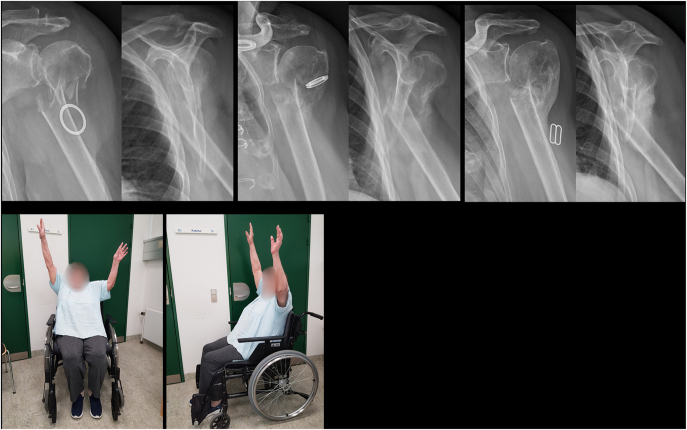
A 79-year-old female with a severely displaced surgical neck fracture. There is no bony contact on the lateral view and limited contact on the anterior–posterior view. The fracture healed with a lateral and posterior bone bridge. Radiographs were taken at admission, 6 weeks, and 6 months. Photographs were taken at 6 months.

Medial and anterior translation of the humeral shaft is another common pattern of displacement in two-part surgical neck fractures. This is a result of the pectoralis major acting on the humeral shaft. Healing appears by lateral and posterior callus bridging (Fig. 5). Reduced pain can be expected when callus bridging is established.

**Figure 6:**
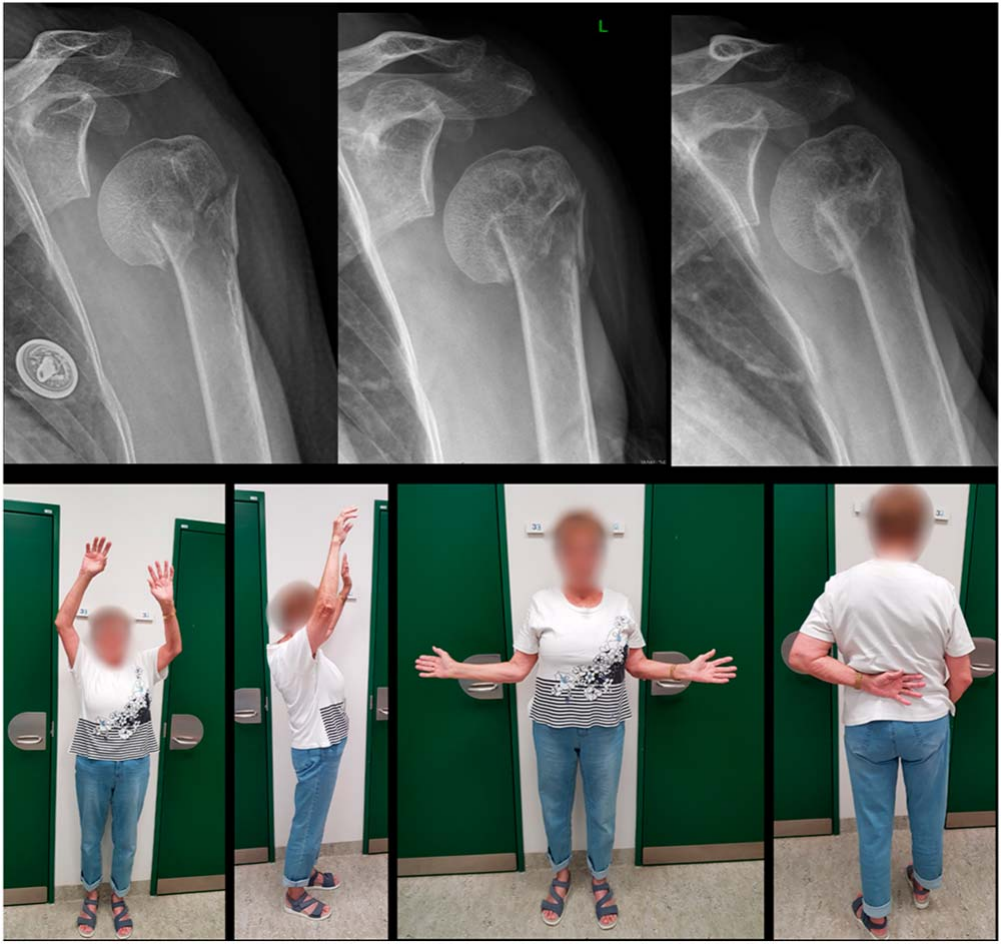
An 80-year-old female presented with a varus-impacted fracture of the surgical neck with a lack of medial support. After 6 weeks, clinical healing was achieved despite incomplete radiographic healing. By 6 months, the humeral head was better centered, and the patient regained pre-injury shoulder function. Anterior–posterior radiographs were taken at 2 weeks, 6 weeks, and 6 months. Photographs were taken at 6 months.

Pain relief and good functional outcome can be obtained following bridging, even with incomplete healing of the medial or anterior part of the fracture complex (Fig. 6). Some degree of angular malunion often remains, but it seems to be well tolerated in the elderly.

## Evidence base

In orthopedic surgery, it is a well-established dogma that increasing fracture displacement is associated with worse clinical outcome and is considered an independent indication for surgical intervention. Recent randomized trials and prospective cohort studies of proximal humeral fractures have challenged this dogma.

Most randomized trials focus on three- and four-part fracture patterns (10). However, two trials have focused on two-part fractures. In a pragmatic British trial (11), 250 patients with displaced fractures were randomized to surgeons’ choice or non-surgical treatment. In 83% of the surgical cases, a locking plate was the implant of choice. Overall, the authors reported no between-group differences in patient-reported shoulder function (Oxford Shoulder Score, OSS) or quality of life (EQ-5D) at 6, 12, 24 months, or 5 years (12). 128 of the 250 patients had Neer two-part surgical neck fractures. A subgroup analysis found no clinically or statistically significant differences between surgically and non-surgically treated patients with Neer two-part fractures (13).

A Nordic trial (14) randomized 88 patients aged 60 or older with two-part Neer surgical neck fractures to locking plate osteosynthesis or non-surgical treatment. After two years, they found no between-group differences in patient-reported upper-limb function (DASH) or quality of life (EQ-5D). They concluded that the usual practice of locking plate osteosynthesis in the elderly may not be beneficial.

Although randomized trials have reported no differences between surgically and non-surgically treated patients, the control groups provide limited data on non-surgical treatment in larger populations. Consecutive, prospective cohort studies from centers treating these fractures non-surgically may help narrow the evidence gap. Outcomes after non-surgical treatment of two-part surgical neck fractures are reported in a few prospective cohort studies.

A prospective cohort of 126 patients (15) with translated two-part fractures (AO A3.2) underwent follow-up at 1 and 5 years. Most patients had more than 66% translation of the humeral shaft. They reported no benefit from surgery regardless of displacement, and they reported no correlation between translation or angulation and return to daily activities. The nonunion rate was 5%; it was not reported whether these patients had clinical symptoms. The same center followed up 99 patients with varus-impacted two-part fractures (AO A2.2) (16). All fractures healed, and 79% had a good-to-excellent outcome (Neer score) after 1 year. They observed that the angular deformity increased in some patients, but they found no correlation between varus angulation and patient outcome. Increasing age was correlated with a worse outcome. Partly overlapping with the above two studies, the authors reported outcomes for 232 patients with Neer two-part surgical neck fractures (17). They found no correlation between final radiographic alignment and patient outcomes.

In a retrospective cohort study examining risk factors for nonunion in adults with proximal humeral fractures treated non-operatively, 2,230 patients were identified from a regional trauma center. Of these, 1,684 patients had fractures involving the surgical neck or below (which also included cases with tuberosity involvement) (18). Cases that were surgically treated (12.6%) were excluded, and patient-reported outcomes were not reported. Nonunion was defined as a lack of clinical or radiographic signs of healing at 24-week follow-up or as the absence of bridging callus on CT in patients experiencing persistent pain. In a multivariate logistic regression analysis, they found that a decreasing head–shaft angle, increasing head–shaft translation, and smoking were independently predictive of the risk of nonunion. The authors recommend that medically fit patients with translated and/or angulated fractures should be considered for surgery to prevent nonunion. However, this recommendation is based on retrospective data and needs supportive data from randomized trials or prospective cohort studies to ensure that unnecessary surgeries are avoided.

In a consecutive cohort of 268 patients with a mean age of 76, Neer two-part surgical neck fractures were followed up for 6 and 12 months (19). After 6 months, they reported a patient-reported shoulder function score (OSS) equivalent to 78% of the maximum score. The expected value in this age group is 82%. Quality of life was measured with EQ-5D. The mean value was 0.79, compared with 0.82 in the background population. Both shoulder function and quality of life improved between 6 and 12 months. The operation rate was 3%.

## Treatment of two-part surgical neck fractures

Proximal humeral fractures in osteoporotic bone may differ from other fractures in that there is no clear correlation between fracture displacement and function or patient satisfaction. Restoring anatomy through surgical reconstruction may appeal to surgeons, but it may not add value to patients with two-part surgical neck fractures compared to non-surgical treatment (20).

In many countries, it remains common practice to perform open reduction and internal fixation in elderly patients with varus-impacted or medially translated two-part surgical neck fractures. A critical aspect of the reconstruction is to achieve medial (or ‘calcar’) support. This approach has been supported by observational studies and the widely held belief that failing to reconstruct the calcar area will lead to clinical failure. Analogies to hip fractures are appealing, despite obvious differences in biomechanics and weight-bearing.

Reports of failed osteosynthesis with screw penetration and cutout into the glenohumeral joint are numerous (21), even from the most esteemed centers (22). The outcome after revision surgery has been disappointing (23).

The failure of locking plates in osteoporotic proximal humeral fractures has not led to the de-implementation of the implant and procedure. Rather, the reflection seems to be: ‘if surgery is not superior to non-surgical treatment, we need to improve the surgery’. Consequently, numerous modifications of the procedure and implant have been proposed, including cement augmentation (24), additional medial plate (25), blade for medial support (26), implants for endosteal support with plates (27, 28), nails (29), stems (30), titanium mesh (31), autologous grafts (32), and allografts (33, 34).

## When is a two-part surgical neck fracture unstable?

Fracture stability is a key concept in orthopedics, and a lack of stability often serves as an independent indication for surgical intervention (35).

Restoring the anatomy has not been shown to be superior to natural healing, despite anterior or medial displacement of the humeral shaft (20). On a popular platform used by most orthopedic residents, it is stated that angularly displaced fractures are most often unstable (36). Non-impacted surgical neck fractures with medial translation are considered unstable and in need of surgery. A threshold for stability is suggested as 33% medial translation of the humeral shaft. The presence of osteoporosis is considered supportive of surgical treatment. The evidence base for these recommendations is weak, and they may lead to unnecessary surgery being continued when taught to young surgeons. Evidence-based and eminence-based approaches to surgical interventions for osteoporotic proximal humeral fractures appear to collide in this clinical question.

The definition of fracture stability by AO may be helpful in the assessment of fractures in weight-bearing bone: A stable fracture is defined as a fracture that does not visibly displace under physiological load  (37).

However, the interpretation in the context of osteoporotic upper-extremity fractures is unclear. What is the physiologic load on the proximal humerus in an 80-year-old female, and how can it be measured? Is the force of gravity a physiological load, a friend, or a foe? Is ‘visible displacement’ during healing a sign of instability, and should it lead to operative intervention? (35).

## Radiographic healing as an indication for late surgery

Delayed union or nonunion is commonly used as an indication for surgical intervention. However, the concepts of delayed union and nonunion are inconsistently used in the scientific literature and in clinical practice. To obtain consensus definitions on radiographic features, a Delphi trial involving 129 international shoulder surgeons was conducted (38). Delayed healing was defined as ‘the absence of bridging callus on at least one of four cortices in the fracture zone on two orthogonal radiographs taken at 3 months after fracture’, and nonunion was defined as ‘the absence of bridging callus on at least one of four cortices in the fracture zone on two orthogonal radiographs taken at 6 months after fracture’.

Although consensus definitions offer advantages, they remain clinically unvalidated. We need more data on radiographic healing, pain, patient-reported shoulder function, and quality of life, and in which cases patients could benefit clinically from late surgery. Imaging and intuitions are unreliable for guiding decisions about surgical intervention in these patients.

In a surgical culture where restoring the anatomy of any displaced long-bone fracture is the benchmark for good practice, it is difficult to predict the outcome of displaced fractures managed without surgery when the patients under study are frail, unfit for surgery, or neglected cases. Treating a healthy elderly patient with a severely displaced surgical neck fracture without surgery makes us uncomfortable and is not considered compatible with good clinical practice. Another hindrance is the limited systematic follow-up using a standardized protocol, ideally by the same healthcare providers. Without access to relevant clinical data, adopting a more evidence-based practice is difficult.

## Concept formation on orthopedics

While the AO definition of stability of an osteosynthesis is relatively clear and known to most orthopedic surgeons, the AO definition of fracture stability is less clear. Defining fracture stability in terms of physiological load does not seem to distinguish between fractures of the proximal humerus and the proximal femur. However, the concept of fracture stability is used in everyday clinical practice without an explicit definition. Based on initial radiographs, some fractures are designated unstable, often leading to surgical intervention. How are these patterns recognized and established?

We learn from our own and others’ experiences. Ostensive identification of fracture patterns requiring surgery is transferred from more experienced to less experienced surgeons. For example, if we encounter a pattern involving displacement of the greater tuberosity, we are likely to infer that the greater tuberosity must be fixed to prevent subacromial impingement. Likewise, if a two-part fracture is medially translated by more than one-third of a bone width, we consider it unstable and a candidate for open reduction and internal fixation to regain calcar support.

A common feature of pattern recognition is that consensus is not based on necessary and sufficient conditions but rather on commonly held, unspoken knowledge or ‘tacit knowledge’. Pattern recognition is widely used in orthopedic decision-making to translate data into classification categories and treatment choices. However, if the data are biased or not informed by the best evidence, they may lead to misleading recommendations and clinical decisions.

If all patients with fractures initially considered unstable are operated on, we will not gain experience with outcomes in patients fit for surgery who are not operated on. Experience with outcomes in patients who are not fit for surgery will introduce bias, as multiple factors other than fracture morphology affect outcomes. For unbiased decision-making, we need data from unselected, consecutive series of relevant patients treated without surgery. Such studies are severely underrepresented in the orthopedic literature. Therefore, we continue to assume that displaced proximal humeral fractures require surgical treatment.

The recognition of patterns in need of surgery may be biomechanically meaningful and align with assessments by more experienced surgeons. This, however, does not necessarily align with best evidence. So far, thirteen randomized trials have found no benefit of surgery for proximal humeral fractures in the elderly (10). Surprisingly, in some Western countries, up to half of these patients are still treated surgically (39, 40). If outcomes after non-surgical management are observed only in patients unfit for surgery, we lose the opportunity to apply the best evidence from randomized trials. The result is unnecessary surgeries.

There is a need for revisiting the central dogma that any displaced long bone fracture must be anatomically reduced and fixed. Considering the best evidence, we must recognize that healing in non-anatomical positions can be compatible with a good patient-reported shoulder function and quality of life, particularly in patients with osteoporotic proximal humeral fractures.

## Implementation of evidence-based practice

How many patients need to undergo surgery to optimize shoulder function and enhance quality of life while avoiding unnecessary and potentially harmful procedures? I suggest following the best available evidence and prioritizing a non-operative approach, while continuing to monitor all patients and remain prepared to convert to surgery if necessary. Primary surgery based on initial imaging is not recommended for surgical neck fractures in elderly patients, except in rare cases of fracture dislocation, neurovascular injury, or risk of skin penetration.

In the emergency room, the patient is given a sling or sling-and-swathe that allows the upper arm to hang freely and benefit from gravity. Adequate pain management is provided according to local guidelines. The patient is informed that evidence does not support primary surgical intervention, but the patient will be carefully monitored, and surgery will be an option if pain persists. The patient is seen in the outpatient clinic after 10–14 days, and exercises are started. Supervised training has not been found superior to careful single instruction (41). The patient is seen again in the outpatient clinic 5–6 weeks after the injury to monitor recovery. If severe pain persists and the patient has a wish for surgery, a reverse shoulder arthroplasty is provided within a few days. It is recommended that the outcomes of all patients, including those treated non-operatively, be measured with appropriate PROMs and quality-of-life assessments at their final visit, usually after 6 weeks.

It is important to note that the follow-up times suggested above are not supported by high-quality evidence. However, clinical outcomes following best evidence have been documented in a prospective cohort of elderly patients with displaced two-part surgical neck fractures, with a surgery rate of 3% (19). The optimal timing for performing a late reverse arthroplasty is a topic of debate and requires a randomized trial to control confounding variables. We recommend conducting the procedure within six weeks, as this timeframe allows for the possibility of tuberosity osteotomy, proper fixation to the stem, and rotator cuff reconstruction.

It is often assumed that the healthiest and most active elderly patients should receive our best treatment, which, for surgeons, usually means surgical intervention. However, this belief is based on a misconception. The healthiest patients tend to have the best outcomes regardless of the treatment they receive. If the healthiest individuals undergo surgery while the less fit do not, it is no surprise that surgery appears to be superior and is deemed appropriate for the healthiest patients. When confounding factors are controlled through randomization, this apparent difference in outcomes disappears.

Patients younger than 60 years have not yet been covered by high-quality evidence. However, this does not necessarily mean that they will benefit from surgery (42). However, a better bone quality may make osteosynthesis a more suitable option.

## Conclusion

- The natural course of healing of osteoporotic proximal humeral fractures often involves changes in fracture morphology.
- We need clinical studies of the association between radiographic appearance and patient-reported outcomes.
- If two-part surgical neck fractures deemed unstable on primary imaging are routinely treated operatively, it will result in overtreatment.
- An evidence-based, non-operative approach should be considered for two-part surgical neck fractures in the elderly.

## ICMJE Statement of Interest

The author declares that there is no conflict of interest that could be perceived as prejudicing the impartiality of the work reported.

## Funding Statement

This work did not receive any specific grant from any funding agency in the public, commercial, or not-for-profit sector.

## Patient consent

All patients have kindly granted permission to publish clinical photos, and their faces have been anonymized.
